# The minimum resting-state fNIRS imaging duration for accurate and stable mapping of brain connectivity network in children

**DOI:** 10.1038/s41598-017-06340-7

**Published:** 2017-07-25

**Authors:** Jingyu Wang, Qi Dong, Haijing Niu

**Affiliations:** 1 State Key Laboratory of Cognitive Neuroscience and Learning & IDG/McGovern Institute for Brain Research, Beijing Normal University, Beijing, 100875 China; 2 Center for Collaboration and Innovation in Brain and Learning Sciences, Beijing Normal University, Beijing, 100875 China

## Abstract

Resting-state functional near-infrared spectroscopy (fNIRS) is a potential technique for the study of brain functional connectivity (FC) and networks in children. However, the necessary fNIRS scanning duration required to map accurate and stable functional brain connectivity and graph theory metrics in the resting-state brain activity remains largely unknown. Here, we acquired resting-state fNIRS imaging data from 53 healthy children to provide the first empirical evidence for the minimum imaging time required to obtain accurate and stable FC and graph theory metrics of brain network activity (e.g., nodal efficiency and network global and local efficiency). Our results showed that FC was accurately and stably achieved after 7.0-min fNIRS imaging duration, whereas the necessary scanning time for accurate and stable network measures was a minimum of 2.5 min at low network thresholds. These quantitative results provide direct evidence for the choice of the resting-state fNIRS imaging time in children in brain FC and network topology study. The current study also demonstrates that these methods are feasible and cost-effective in the application of time-constrained infants and critically ill children.

## Introduction

Resting-state functional near-infrared spectroscopy (fNIRS) is an emerging area of interest and is currently attracting increasing attention as a promising imaging tool for the study of resting-state brain function^1^. By measuring the brain’s low-frequency concentration fluctuation of hemoglobin, fNIRS provides chances to explore functional interactions between segregated brain regions in the resting brain. Such functional interactions are defined as resting-state functional connectivity (FC) and are reported to form resting-state networks. A wealth of research has demonstrated that human functional brain networks can be constructed using resting-state fNIRS imaging data^1, 2^.

As a newly developed optical imaging tool, fNIRS uses light in the near-infrared spectrum (670–900 nm) to noninvasively monitor hemodynamic responses evoked by brain activity and to obtain quantitative concentration changes in two chromophores of oxygenated hemoglobin (HbO) and deoxygenated hemoglobin (HbR) in tissue^1, 3^. Relative to the widely used functional magnetic resonance imaging (fMRI), the fNIRS technique displays some unique advantages such as high portability, quietness, data acquisition in a natural environment and high robustness to head motion^4^. These special advantages are facilitating the use of fNIRS as a great potential tool for human brain function studies in children with normal neural development or a clinical diseased state^5^. Specifically, recent advances in main brain regions (e.g., frontal, temporal, parietal and occipital lobes of the cerebral cortex) data acquisition have allowed fNIRS to construct entire cortical connectivity networks and obtain the topological organizational features of the constructed brain network. These quantitative brain connectivity and topological properties have been found to change during normal development^6^ or in the context of diseases^5, 7–9^, enhancing our understanding of organizational principles of central nervous system development in healthy and diseased populations.

However, to facilitate the use of the fNIRS-based imaging technique in the study of brain networks in children, one critical step is to determine the minimum data acquisition duration that is capable of providing accurate and stable functional brain connectivity and graph theory metrics. Regrettably, the current resting-state fNIRS field is lacking such evidence, and the general scanning duration for the calculation of brain connectivity networks in children is approximately 2.0–20.0 min^8^. An overly short fNIRS imaging data acquisition duration will possibly decrease the accurate characterization of the brain connectivity network, whereas a long duration for data acquisition is commonly problematic and a challenge for infants and young normal or critically ill children. However, how the duration of fNIRS signal scanning is related to stable functional brain connectivity and graph theory metrics of the brain connectivity network remains largely unknown. The empirical conclusions will provide critical information for the brain connectivity network in children with normal brain development and the clinical implementation of the fNIRS technique.

In the present study, functional brain network connectivity and graph theoretical analyses were applied to a series of incrementally longer temporal windows of resting-state fNIRS imaging data. We hypothesized that functional brain connectivity and the corresponding network metrics would stabilize after a certain number of expanding windows, requiring different durations of resting-state fNIRS imaging signal acquisition for optimal characterization. Here, fNIRS data were collected from 53 healthy children at their resting state. We evaluated the influence of fNIRS imaging time on the accuracy and stability of brain functional connections and graph theory metrics of the brain connectivity network.

## Results

### FC maps

**Figure 1:**
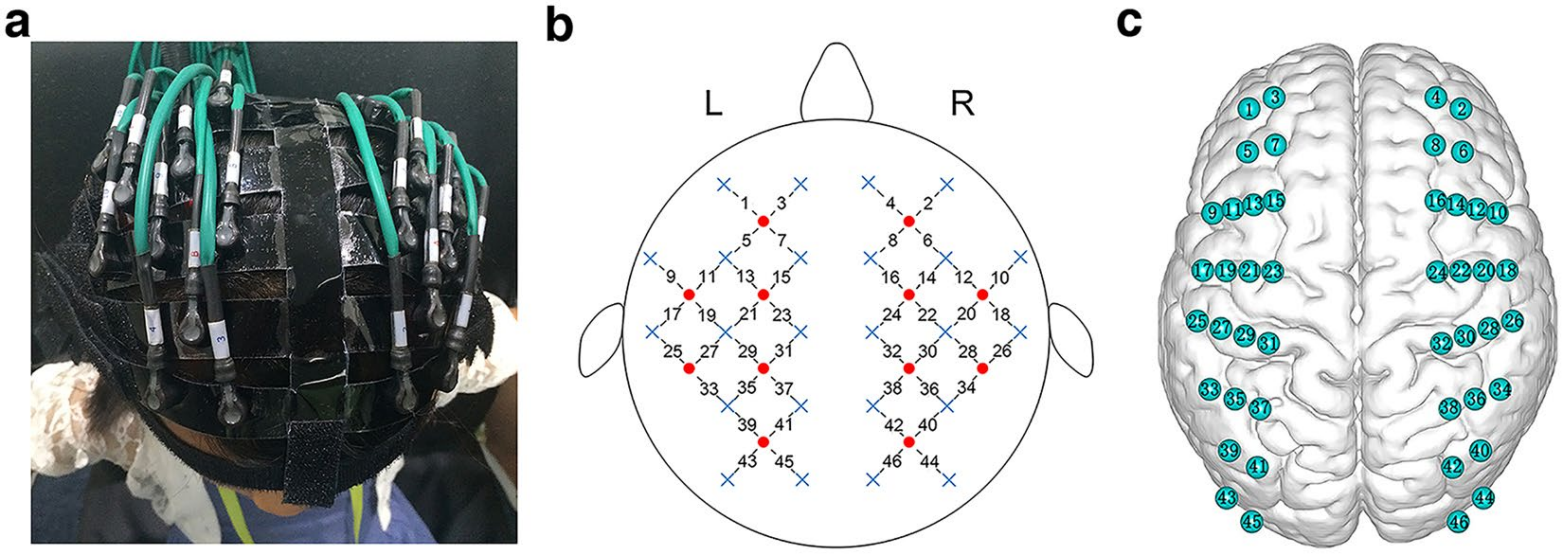
Schematic of fNIRS channel localization. (**a**) Photograph of fNIRS measurement of a participant. (**b**) The schematic of the imaging pad (12 sources, red circle and 24 detectors, blue cross). The sources and detectors were symmetrically placed on the left and right hemispheres and constituted 46 measurement channels, which allowed for the most brain regions (i.e., frontal, temporal, parietal, and occipital lobes) on two half-hemispheres to be measured. (**c**) The anatomical position of each measurement channel.

**Figure 2:**
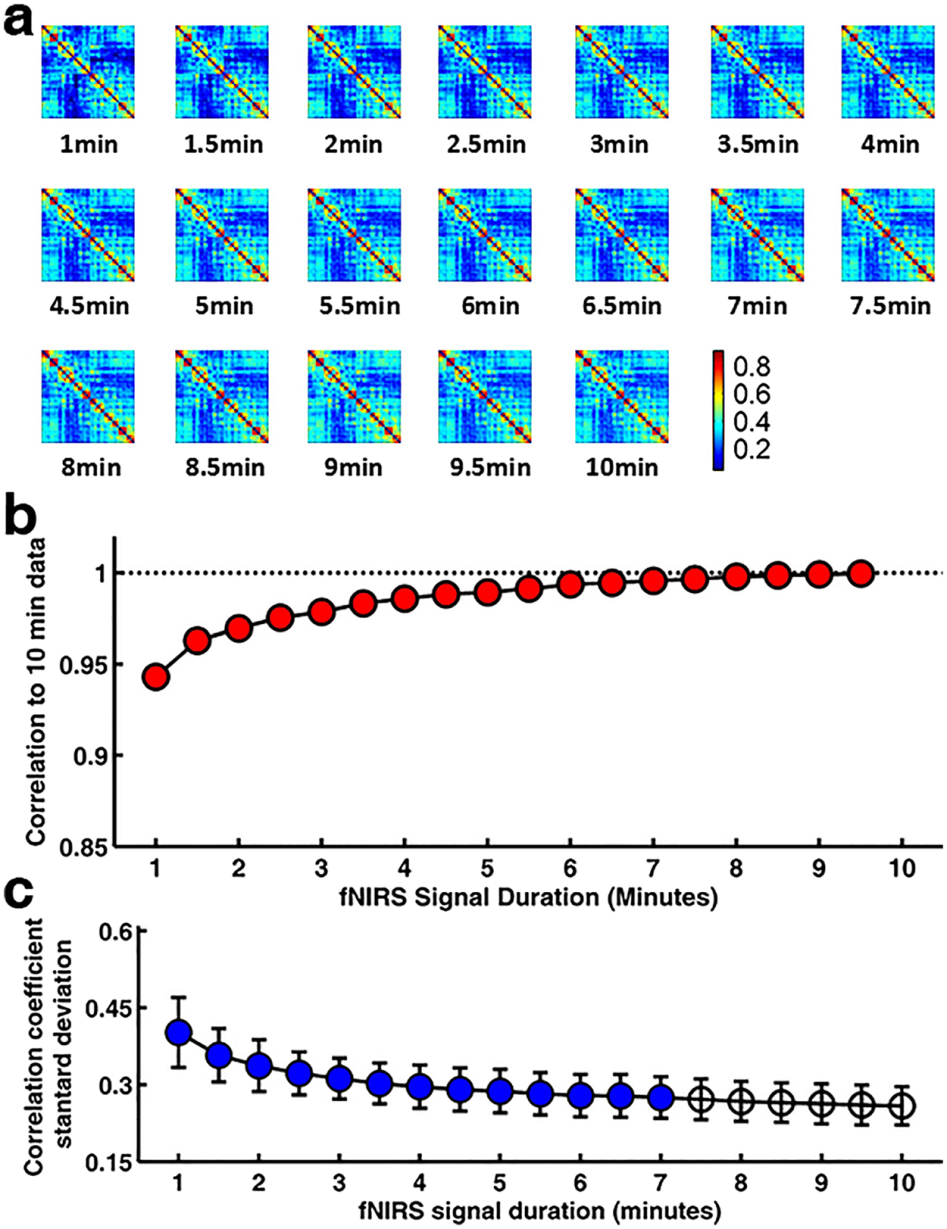
Effect of the fNIRS signal acquisition duration on the accuracy and stability of the spatial FC pattern. (**a**) The FC maps calculated by Pearson correlation for fNIRS signal acquisition durations ranging from 1.0 to 10 min with 30-sec bins. Visually, the spatial patterns of these FC maps exhibited high similarity across variable data collection lengths. (**b**) Accuracy curve. The plots of the correlation strength calculated for the spatial patterns of FC maps (transferred to z score before calculation) between short and long (10.0 min) signal durations. The red-filled circles indicate significant correlation of the spatial FC maps associated with a given fNIRS signal acquisition duration with the FC map when computed using 10.0 min of fNIRS data. The analysis revealed significant (*P* < 0.001) and strong correlations for all acquisition durations, indicating almost immediate accuracy of the FC maps. (**c**) Stability curve. The plots of the correlation coefficient associated with the standard deviation (mean ± SD) plotted according to the duration of the fNIRS signal acquisition (1.0~10.0 min in 30-sec bins). The blue-filled circles indicate a significant difference in the magnitude of the computed value associated with a given fNIRS signal acquisition duration compared with the magnitude when computed using 10.0 min of fNIRS data. The magnitude of the correlation coefficient standard deviation decreased as the fNIRS signal acquisition duration increased, with little change in magnitude after approximately 7.0 min of data collection.

For each participant, we measured hemodynamic signal changes from multiple regions of the cerebral cortex (Fig. 1) and then calculated the individual FC at different fNIRS signal acquisition durations. Figure 2a shows the group-level FC maps associated with an increasing fNIRS signal acquisition duration, which were calculated by averaging all of the functional correlation matrixes for every acquisition duration across subjects. Visually, the spatial patterns of these FC maps exhibited high similarity across the variable data collection lengths. Quantitatively, when compared to the relatively longer 10.0-min data acquisition duration, these results revealed a significant (*P* < 0.001) and strong correlation across each fNIRS signal duration (the mean correlation coefficient *r* = 0.98 ± 0.03; Fig. 2b). This suggests that the short fNIRS signal acquisition duration, i.e., 1.0 min, can result in FC maps with as high an accuracy as those calculated with a 10.0-min scanning duration. However, in respect to FC stability, the statistical analysis of the correlation coefficient SD (one-way ANOVA and Dunnett post hoc analysis) showed that FC maps computed using an fNIRS signal acquisition duration longer than 7.0 min were no different than those computed using a 10.0-min fNIRS data acquisition duration (Fig. 2c, Table 1). This indicated that FC remained stable only when the fNIRS imaging data acquisition duration was sufficiently long, e.g., ≥7.0 min.

**Table 1:** Dunnett Post Hoc Analysis Data for Measures of Correlation Coefficient Stability Associated with fNIRS Signal Acquisition Duration.

	1.0 minute fNIRS Signal Acquisition	1.5 minute fNIRS Signal Acquisition	2.0 minute fNIRS Signal Acquisition	2.5 minute fNIRS Signal Acquisition	3.0 minute fNIRS Signal Acquisition	3.5 minute fNIRS Signal Acquisition	4.0 minute fNIRS Signal Acquisition
P	<0.001 (0.1266, 0.1598)	<0.001 (0.0819, 0.1151)	<0.001 (0.0618, 0.0949)	<0.001 (0.0467, 0.0799)	<0.001 (0.0365, 0.0697)	<0.001 (0.0271, 0.0603)	<0.001 (0.0207, 0.0539)
MD	0.1432 (0.0101)	0.0985 (0.0088)	0.07835 (0.0086)	0.0633 (0.0077)	0.0531 (0.0075)	0.0437 (0.0075)	0.0373 (0.0078)
	4.5 minute fNIRS Signal Acquisition	5.0 minute fNIRS Signal Acquisition	5.5 minute fNIRS Signal Acquisition	6.0 minute fNIRS Signal Acquisition	6.5 minute fNIRS Signal Acquisition	7.0 minute fNIRS Signal Acquisition	7.5 minute fNIRS Signal Acquisition
P	<0.001 (0.0151, 0.0483)	0.001 (0.0120, 0.0452)	0.005 (0.0070, 0.0402)	0.018 (0.0035, 0.0367)	0.022 (0.0029, 0.0361)	0.044 (0.0003, 0.0329)	0.133
MD	0.0317 (0.0078)	0.0286 (0.0078)	0.0236 (0.0077)	0.0201 (0.0077)	0.0195 (0.0077)	0.0163 (0.0076)	0.0127 (0.0075)
	8.0 minute fNIRS Signal Acquisition	8.5 minute fNIRS Signal Acquisition	9.0 minute fNIRS Signal Acquisition	9.5 minute fNIRS Signal Acquisition	10.0 minute fNIRS Signal Acquisition		
P	0.303	0.452	0.629	0.841	>0.99		
MD	0.0087 (0.0075)	0.0064 (0.0074)	0.0041 (0.0075)	0.0017 (0.0075)	0		

Note—Dunnett post hoc analysis data using the longest fNIRS signal acquisition duration (10.0 min) as the control group for pairwise comparisons. Data are *P* values (data in parentheses are 95% CIs) and Mean Difference (STD Error).

### Network nodal efficiency

**Figure 3:**
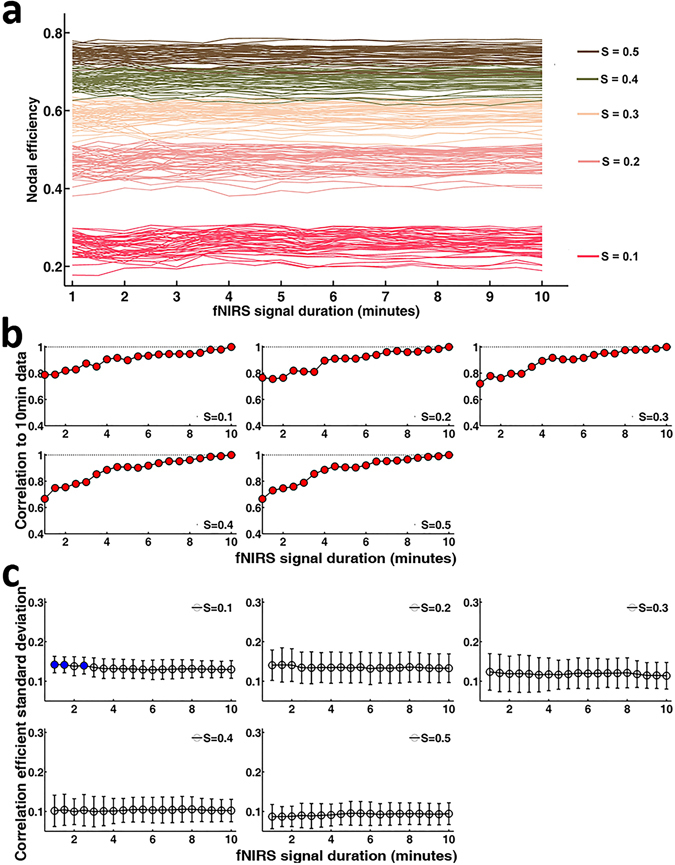
Effect of fNIRS signal acquisition duration on the accuracy and stability of nodal efficiency. (**a**) Magnitude of nodal efficiency at different sparsity values (0.1, 0.2, 0.3, 0.4 and 0.5) plotted according to the duration of the fNIRS signal acquisition ranging from 1.0 to 10.0 min. Curves with the same color represent measurement channels from 1 to 46. Different colors represent nodal efficiency calculated at different sparsity threshold values. (**b**) The plots of the correlation strength calculated for the spatial pattern of nodal efficiency between short and long (10.0 min) signal durations. The red-filled circles indicate a significant correlation of the nodal efficiency map associated with a given fNIRS signal acquisition duration with the nodal efficiency computed using 10.0 min of fNIRS data. The analysis revealed significant (*P* < 0.001) and strong correlations for all threshold conditions, indicating almost immediate accuracy of the nodal efficiency. (**c**) The plots of the nodal efficiency-associated standard deviation (mean ± SD) plotted according to the duration of the fNIRS signal acquisition (1.0–10.0 min in 30-sec bins). The blue-filled circles indicate a significant difference in the magnitude of the computed value associated with a given fNIRS signal acquisition duration compared with the magnitude when computed using 10.0 min of fNIRS data. These data approximately correspond to horizontal lines, with little difference between the magnitudes of the computed graph metrics for each fNIRS collection duration.

The plots of nodal efficiency (Fig. 3a) showed approximately horizontal lines with little difference between the magnitudes of measured values across the scanning duration for each sparsity threshold value, and the magnitudes of the nodal efficiency showed obvious increases with sparsity threshold values. For evaluation of nodal efficiency accuracy, the correlation analysis of network measures calculated between the short and the relatively long 10.0-min data acquisition duration revealed significant (*P* < 0.001) and strong correlations (Fig. 3b) for all threshold conditions, indicating an almost immediate accuracy of the nodal efficiency.

For the evaluation of nodal efficiency stability, statistical analysis (two-way ANOVA) revealed no significant interaction between the fNIRS signal acquisition duration and sparsity (*F* [72, 4275] = 0.613, *P* = 0.996, partial η^2^ = 0.010), but there was a significant but relatively small main effect of the fNIRS signal acquisition duration on the magnitude of nodal efficiency (*F* [18, 4275] = 1.636, *P* = 0.044, partial η^2^ = 0.007). Dunnett post hoc analyses further revealed significant differences associated with the fNIRS signal data collection duration only at a sparsity of 0.1, as follows: There was a significant 6.3% difference (95% confidence interval [CI]: −0.0652, −0.0094; *P* = 0.005) between the magnitude of nodal efficiency computed using 10.0 min and that using 1.0 min of fNIRS data, a significant 3.3% difference (95% confidence interval [CI]: −0.0387, −0.0021; *P* = 0.036) between that computed using 10.0 min and 1.5 min of fNIRS data, and a significant 6.6% difference (95% confidence interval [CI]: −0.0684, −0.0064; *P* = 0.005) between that computed using 10.0 min and 2.5 min of fNIRS data (Fig. 3c).

### Network global efficiency

**Figure 4:**
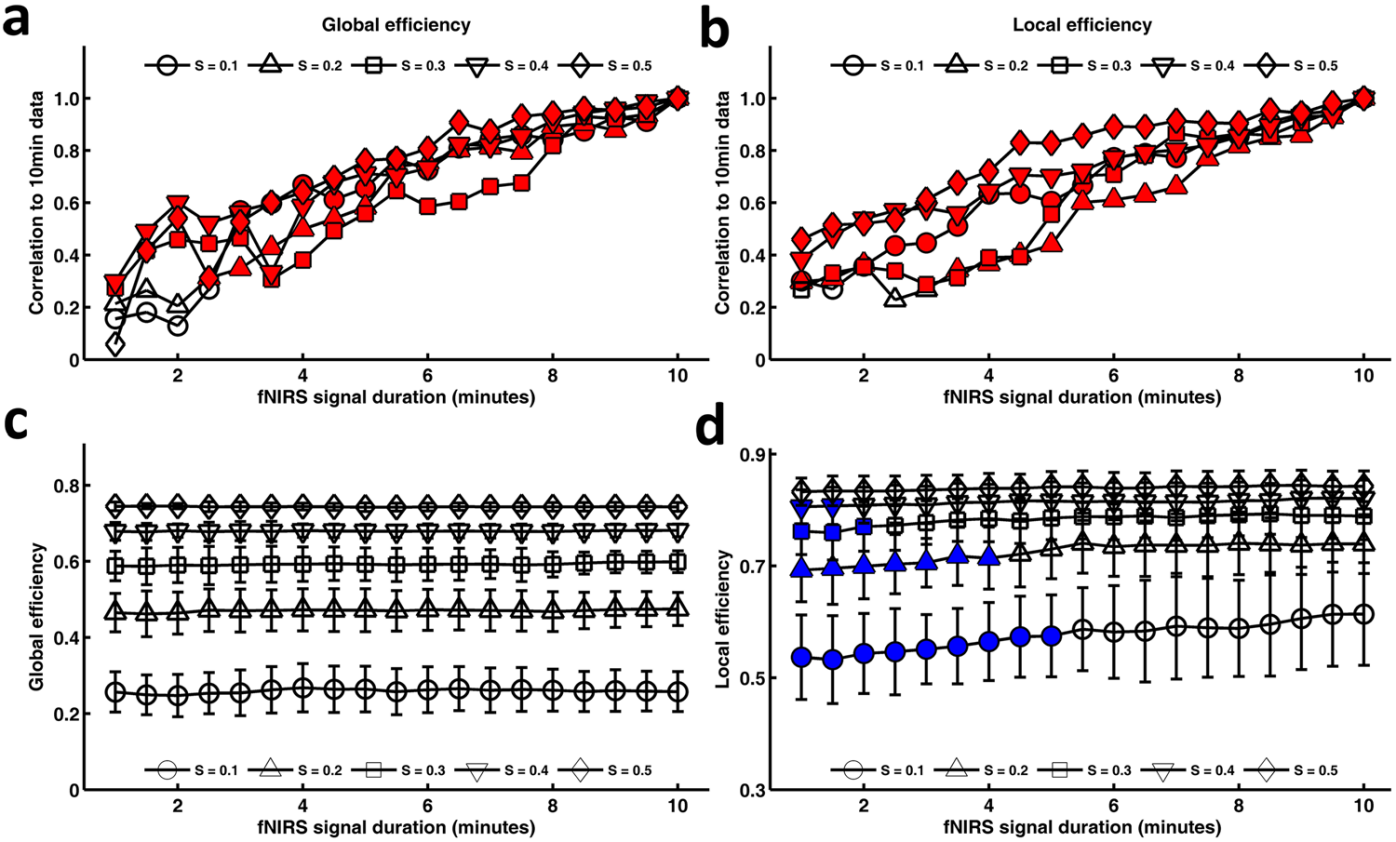
Effect of fNIRS signal acquisition duration on the accuracy and stability of global efficiency and local efficiency. The plots of the correlation strength calculated for global efficiency (**a**) and local efficiency (**b**) between short and long (10.0 min) signal durations. The red-filled circles indicate a significant correlation between global/local efficiency calculated with short and long (10.0 min) fNIRS signal acquisition durations. For both global and local efficiency, the analyses revealed significant (*P* < 0.001) and strong correlations for almost all threshold conditions. Magnitudes of the global efficiency (**c**) and local efficiency (**d**) plotted according to the duration of the fNIRS signal acquisition (1.0~10.0 min with 30-sec bins). The blue-filled shapes indicate a significant difference in the magnitude of the computed value associated with a given fNIRS signal acquisition duration compared with the magnitude when computed using 10.0 min of fNIRS data.

For the evaluation of global efficiency accuracy, the correlation analysis of network measures calculated between the short and the relatively long 10.0-min data acquisition duration also revealed significant (*P* < 0.001) and strong correlations for several threshold conditions, such as 0.3 and 0.4 (Fig. 4a), which indicated an almost immediate accuracy of the network global efficiency. However, when the global efficiency was calculated at sparsity threshold values of 0.1 and 0.2, the correlation of the global efficiency calculated between the short and the relatively long 10.0-min data acquisition duration was significant only for scanning durations longer than 3.0 min and 2.5 min, respectively.

For the evaluation of global efficiency stability, the two-way ANOVA revealed no significant interaction between the fNIRS signal acquisition duration and sparsity (*F* [72, 4940] = 0.254, *P* = 1.000, partial η^2^ = 0.004), and there was also no significant main effect of the fNIRS signal acquisition duration on the magnitude of the global efficiency at any network sparsity threshold value (*F* [18, 4940] = 0.676, *P* > 0.050, partial η^2^ = 0.002) (Fig. 4c). This indicated that global efficiency values computed using 1.0 min of fNIRS signal acquisition were no different than those computed using 10.0 min of fNIRS data acquisition.

### Network local efficiency

Similar to global efficiency, the correlation analysis of network local efficiency calculated between the short and the relatively long 10.0-min data acquisition duration also revealed significant (*P* < 0.001) and strong correlations for almost all threshold conditions (Fig. 4b), indicating an almost immediate accuracy of the network local efficiency. Meanwhile, the local efficiency was noted to be accurate when the scanning duration was longer than 1.5 min and 3.0 min at the sparsity threshold conditions of 0.1 and 0.2, respectively.

For the evaluation of local efficiency stability, the two-way ANOVA revealed a significant interaction between the fNIRS signal acquisition duration and sparsity (*F* [72, 4940] = 1.903, *P* < 0.001, partial η^2^ = 0.027). The simple effect test found that the main effect of the fNIRS signal acquisition duration was significant at a sparsity of 0.1 (*F* [18, 4998] = 2.91, *P* < 0.001). The longer the acquisition duration, the greater the local efficiency value. However, at a sparsity of 0.5, the main effect of the fNIRS signal acquisition duration was not significant, *F* < 1. Dunnett post hoc analyses further revealed significant differences associated with the fNIRS signal data collection duration at sparsities of 0.1, 0.2, 0.3 and 0.4, as follows: At a sparsity of 0.1, there was a significant 6.4–12.6% difference between the magnitude of local efficiency calculated using 10.0 min of data and that using short acquisition durations ranging from 1.0 to 5.0 min (Fig. 4d, Table 2). At a sparsity of 0.2, there was a 3.4–7.6% significant difference between that calculated using 10.0 min of data and that using short acquisition durations ranging from 1.0 to 4.0 min (Fig. 4d, Table 2). At a sparsity of 0.3, there was a 2.4–3.4% significant difference between that calculated using 10.0 min of data and that using short acquisition durations ranging from 1.0 to 2.0 min (Fig. 4d, Table 2). At a sparsity of 0.4, there was a 1.6–1.8% significant difference between that calculated using 10.0 min of data and that using short acquisition durations ranging from 1.0 to 1.5 min (Fig. 4d, Table 2). Table 2 didn’t show the *P* values of the differences after 5.0 min of data acquisition because they were all not significant at these sparsity threshold values.

**Table 2:** Dunnett Post Hoc Analysis Data for Local Efficiency Stability Associated with fNIRS Signal Acquisition Duration.

Sparsity		1.0 minute fNIRS Signal Acquisition	1.5 minute fNIRS Signal Acquisition	2.0 minute fNIRS Signal Acquisition	2.5 minute fNIRS Signal Acquisition	3.0 minute fNIRS Signal Acquisition
0.1	P	<0.001 (−0.1124, −0.0505)	<0.001 (−0.1124, −0.0504)	<0.001 (−0.1015, −0.0040)	<0.001 (−0.0984, −0.0364)	<0.001 (−0.0938, −0.0318)
	MD	−0.0771 (0.0163)	−0.0814 (0.0166)	−0.0705 (0.0160)	−0.0674 (0.0164)	−0.0628 (0.0152)
0.2	P	<0.001 (−0.0673, −0.0256)	<0.001 (−0.0643, −0.0225)	<0.001 (−0.0606, −0.0189)	0.001 (−0.0568, −0.0150)	0.002 (−0.0543, −0.0125)
	MD	−0.0464 (0.0106)	−0.0434 (0.0114)	−0.0397 (0.0108)	−0.0359 (0.0102)	−0.0334 (0.0094)
0.3	P	0.001 (−0.0426, −0.0114)	<0.001 (−0.0455, −0.0143)	0.018 (−0.0345, −0.0033)	0.057	0.139
	MD	−0.0270 (0.0082)	−0.0299 (0.0088)	−0.0189 (0.0084)	−0.0166 (0.0085)	−0.0118 (0.0079)
0.4	P	0.021 (−0.0274, −0.0023)	0.044 (−0.0255, −0.0004)	0.062	0.112	0.075
	MD	−0.0149 (0.0062)	−0.0129 (0.0065)	−0.0120 (0.0063)	−0.0102 (0.0065)	−0.0114 (0.0065)
0.5	P	0.053	0.119	0.094	0.118	0.204
	MD	−0.0098 (0.0050)	−0.0081 (0.0052)	−0.0087 (0.0053)	−0.0082 (0.0052)	−0.0066 (0.0052)
Sparsity		3.5 minute fNIRS Signal Acquisition	4.0 minute fNIRS Signal Acquisition	4.5 minute fNIRS Signal Acquisition	5.0 minute fNIRS Signal Acquisition	
0.1	P	<0.001 (−0.0884, −0.0264)	0.002 (−0.0801, −0.0181)	0.010 (−0.0715, −0.0095)	0.013 (−0.0701, −0.0081)	
	MD	−0.0574 (0.0156)	−0.0491 (0.0158)	−0.0405 (0.0160)	−0.0391 (0.0161)	
0.2	P	0.043 (−0.0424, −0.0006)	0.018 (−0.0461, −0.0044)	0.089	0.395	
	MD	−0.0215 (0.0102)	−0.0253 (0.0105)	−0.0181 (0.0108)	−0.0091 (0.0102)	
0.3	P	0.373	0.562	0.270	0.651	
	MD	−0.0071 (0.0078)	−0.0046 (0.0081)	−0.0088 (0.0080)	−0.0036 (0.0079)	
0.4	P	0.259	0.293	0.506	0.487	
	MD	−0.0072 (0.0065)	−0.0067 (0.0063)	−0.0043 (0.0064)	−0.0045 (0.0065)	
0.5	P	0.333	0.482	0.484	0.849	
	MD	−0.0050(0.0051)	−0.0037(0.0052)	−0.0036(0.0051)	−0.0010(0.0053)	

Note—Dunnett post hoc analysis data using the longest fNIRS signal acquisition duration (10.0 min) as the control group for pairwise comparisons. Data are *P* values (data in parentheses are 95% CIs) and Mean Difference (STD Error).

## Discussion

Evidence has shown that functional brain connectivity and the network topological features are highly dynamic even in the resting brain^10, 11^. Image acquisition protocols that minimize the temporal variability of connectivity patterns and network topological architectures can facilitate the discovery of imaging biomarkers in both healthy and diseased children. This is the first study to demonstrate the effect of resting-state fNIRS scanning duration on functional brain connectivity and graph theoretical metrics in data from children, as assessed by accuracy and stability when comparing these metrics calculated from short and relatively longer 10.0-min data acquisition durations. We found that accuracy of FC was achieved after 1.0 min of fNIRS imaging duration (Fig. 2b), whereas the FC was stabilized after 7.0 min of fNIRS imaging acquisition (Fig. 2c). As such, to obtain both accurate and stable FC requires a minimum of 7.0 min of resting-state fNIRS data acquisition. For accurate and stable network measures, we found that the necessary scanning time of resting-state fNIRS data was a minimum of 2.5 min at low network thresholds (Figs 3b,c and 4). These findings are consistent with our hypothesis that the functional brain connectivity networks become stable along increasing temporal trajectories, and they suggest that different resting-state fNIRS scanning durations may be used depending on the outcome of interest. Although these data were consistent with our proposed hypothesis, the magnitude of the difference in the fNIRS collection duration required for data stability between FC (Fig. 2c) and graph theory metrics (Figs 3c and 4c,d) showed obvious differences. Furthermore, our findings also showed good compatibility with the previous fMRI results^12, 13^ that demonstrated the scanning time required to achieve stable FC was longer (5.0 min) than that required to achieve stable topological metrics of the brain network (e.g., 2.0 min of fNIRS signal acquisition for local efficiency and global efficiency). One possible explanation for the relatively rapid stabilization of the graph theory data is that the intrinsic relationships between the regional cerebral regions that underlie the graph theory output are present within the connectivity network at the earliest time points^12^. It was also possible that the signal used to calculate the graph metrics might be much higher than the noise within the connectivity network across all data collection durations. Certainly, further studies based on large samples of data sets from children are expected to provide valid evidence in the future on the temporal dynamics underlying the differences in stability between FC and network metrics.

In the current study, we adopted several specific network sparsity threshold values (e.g., from 0.1 to 0.5 with an interval of 0.1) to construct child brain networks and calculate specific network measures. Human brain functional networks are known to exhibit economical small-world properties at low threshold conditions^14, 15^. As such, the application of low network threshold values in our study was useful since it was able to allow human brain network properties, such as small-world properties, to be properly estimated and guaranteed that the number of spurious edges in each network was minimized. Meanwhile, the choice of threshold approach in our study also provided convenient comparisons of data across individual subjects by constructing comparable networks of equivalent size. Notably, there is currently no definitive way to select a single threshold during the construction of brain network, and a general strategy is to threshold each correlation matrix repeatedly using several threshold values (e.g., 3%, 5% and 7% in the study by Cao *et al*.^16^ and 1%, 5%, 10% and 20% in the study by Dai *et al*.^17^) or over a range of sparsity values (e.g., 6% ≤ sparsity ≤ 40% in the study by He *et al*.^18^ and 5% ≤ sparsity ≤50% in the study by Martijn *et al*.^19^) and then estimate the properties of the resulting graphs at each threshold value. In the current study, nodal efficiency and network local and global efficiency increased as the network sparsity threshold values increased. The magnitude of nodal efficiency and network local and global efficiency at each network sparsity threshold value was similar to values in those prior studies in which these graph theory metrics were used^12^. Specifically, for nodal efficiency and network global efficiency, our results (Figs 3 and 4) demonstrated that the effects of the fNIRS data collection duration on the magnitude of efficiency were independent of the network sparsity threshold values that were adopted in the study. As such, the network sparsity threshold values from 0.1 to 0.5 might be an appropriate choice to be applied in future studies on children in which graph theoretical analyses are utilized. However, the results of the local efficiency analyses showed a relatively larger dependence on network sparsity threshold values, e.g., the minimum fNIRS scanning duration to reach stable local efficiency decreased from 5.0 min to 1.5 min with the increasing of network sparsity threshold values from 0.1 to 0.4 (Fig. 4d). As such, network sparsity threshold values larger than 0.4 might be appropriate for local efficiency analysis in studies on children in the future when considering a relatively shorter fNIRS data scanning duration. However, further studies are required to determine the optimal network sparsity threshold values for examining the brain networks of clinical children using fNIRS data.

A few issues need to be further addressed. First, the sources and detectors used in the current study were only placed on main brain regions (e.g., frontal, temporal, parietal and occipital lobes of the cerebral cortex) on bilateral hemispheres, which limited the whole-brain network analysis performed by fNIRS and further inhibited the evaluation of topological connectivity among multiple cortical neural systems. Second, the current methodology for processing fNIRS signals is far from standardized^20^, especially in regard to removing effects of typical noise sources, e.g., motion artifacts. However, head motion noise is considered to be a primary noise source in data from children. Although the ICA approach was adopted in our study to remove the typical noise component in the hemoglobin signals, whether there were potential noise components contaminating the accurate evaluation of the fNIRS scanning time on FC and network metrics remained undetermined. Certainly, the other technical details involved in fNIRS brain network construction could also affect the network properties and their accuracy or stability characterization; for example, whether a global signal regression exists and how to select the frequency band during preprocessing is beyond the scope of this paper and is worth conducting separate studies in the future. Third, we only evaluated three commonly used network measures associated with increasing fNIRS collection durations, and thus, whether the current findings were valid for other network metrics remains largely unknown. Fourth, we observed temporal stability of graph metrics based on several specific chosen sparsity threshold values, but it would also be interesting to introduce some objective methods for network sparsity threshold selection to further evaluate the effect of fNIRS scanning time on the accuracy and stability of graph metrics. Finally, the resting-state fNIRS imaging data used in this study were from healthy child participants. As such, the current results have not been validated in children with neurologic or psychiatric abnormalities, where immaturity in the brain and abnormalities of the central nervous system in those children may change the current conclusion of the FC and network efficiency evaluation, possibly requiring longer fNIRS data collection durations. Therefore, exploring possible specificities for such populations with respect to specific fNIRS imaging durations is necessary, which may have important implications in the application of network analyses in healthy and diseased children.

In summary, for studies of children, as little as 1.0 min of resting-state fNIRS imaging signals may be sufficient to obtain accurate FC and both accurate and stable graph theory metrics for dynamic brain network analysis. This finding indicates that it is feasible and cost-effective to apply these methods to brain imaging studies for normal and disease-associated brain development, even in populations in which routine imaging is highly challenging, such as infants and critically ill children.

## Materials and Methods

### Participants and Protocol

Fifty-three healthy right-handed children (38 males; age range: 6.9–8.21 years, mean age 7.38 ± 0.34 years) participated in this study. Written informed consent was obtained from each child and his/her parents prior to the experiment. Data collection was carried out according to the protocols approved by the Review Board at the State Key Laboratory of Cognitive Neuroscience and Learning, Beijing Normal University. The time duration for resting-state fNIRS data acquisition was approximately 11.0 min for each subject. During the scanning, the subjects were required to relax and remain still with their eyes closed without falling asleep.

### Data acquisition

A continuous-wave near-infrared optical imaging system (CW6, TechEn Inc., MA, USA) was used to measure the time course of HbO and HbR concentrations at a sampling rate of 50 Hz. The system included 12 laser sources and 24 detectors, with each source including two wavelengths (690 and 830 nm) of near-infrared light^21, 22^. The sources and detectors were systematically placed on the left and right hemispheres of the participant’s head, with spatial separation between adjacent sources and detectors at 3.2 cm. This probe design was also exactly the same as that in our previous series of fNIRS studies^1, 3, 23^. The configuration resulted in 46 measurement channels that covered the frontal, temporal, parietal and occipital lobes (Fig. 1) of the cerebral cortex. The positions of the probes were determined according to the international 10–20 system of electrode placement, and the external auditory canals and vertex of each participant were referenced as landmarks. Specifically, the detectors below channels 17 to 24 in both hemispheres were set along a coronal line from the vertex to the external auditory pores; thus, their midline was localized in Cz, and the leftmost and rightmost detectors were fitted around T3 and T4, respectively.

### Data preprocessing

We used the modified Beer-Lambert law (MBLL)^24^ to calculate changes in hemoglobin concentrations from the attenuation of light entering the head at two wavelengths. The time course of the hemoglobin concentration was subsequently subjected to a temporal ICA analysis to remove typical motion-induced artifacts and systematic noise and was further band-pass filtered (0.01~0.1 Hz) to obtain low frequency hemodynamic fluctuations^25–27^. To examine the effect of the resting-state scanning duration on functional brain connectivity and topological network metrics, we truncated the motion-corrected 10-min data into 30-second time epochs that ranged from 1.0 to 10.0 min.

### Functional Network Connectivity and Graph Theoretical analysis

#### Functional connectivity (FC) definition

In fNIRS study, the nodes were defined as measurement channels, and edges were defined as functional connectivity between nodes. Functional connectivity was quantified by computing Pearson correlation coefficients for the hemoglobin concentration time series between any two nodal brain regions. For an arbitrary given participant, the Pearson correlation was separately calculated to generate a 46 × 46 correlation matrix at each acquisition duration.

#### Network thresholding

To obtain a binarized network, each correlation matrix was thresholded into a binarized matrix with a fixed sparsity threshold value, which is also similar to the process performed in our previous studies^1, 3^ that defined sparsity as the number of existing edges divided by the maximum possible number of edges within a network. Because there is currently no definitive way to select a single threshold, we thresholded each correlation matrix repeatedly over a wide range of sparsity values (0.1~0.5) and then estimated the properties of the resulting graphs at each threshold value. The range of sparsity values was chosen here to allow small-world network properties to be properly estimated and the number of spurious edges in each network to be minimized as indicated in previous studies^28^.

#### Network measures

In graph theory, network efficiency has often been proposed to describe the information communication ability within a network^29, 30^. The corresponding network efficiency metrics have also generally been found to characterize normal brain development^16, 31, 32^ and some clinically related brain diseases^33–36^ and have been confirmed to have numerous conceptual and technical advantages^14, 37^. We, therefore, chose these measures as the network measures of interest when assessing the optimal fNIRS data acquisition duration. Specifically, nodal efficiency, network global efficiency and local efficiency were separately calculated for each of the constructed networks using different fNIRS data acquisition durations. Of note, an in-house FC-NIRS package^38^ was adopted for the network metric calculation. Specifically, the definitions of these efficiency metrics are summarized as follows:

#### Nodal efficiency

Nodal efficiency (*E*_*nodal*_) characterizes the capacity of a node to exchange messages with the other nodes of the network *G*, and it is generally defined as follows:

1 $${E}_{nodal}(i)=\frac{1}{N-1}\sum _{i\ne j\in G}\frac{1}{{d}_{ij}}$$

where *d*_*ij*_ is the shortest path length between node *i* and node *j*, and *N* is the quantity of nodes in the network.

#### Network global efficiency

Global efficiency is a global metric that characterizes the ability to transfer information in the entire brain network, and it is computed as the mean of nodal efficiency across all nodes of the network^29^:

2 $${E}_{glob}(G)=\frac{1}{N(N-1)}\sum _{j\ne i\in G}\frac{1}{{d}_{ij}}$$

where *d*_*ij*_ is the shortest path length between node *i* and node *j*, and *N* is the quantity of nodes in the network.

#### Network local efficiency

Network local efficiency characterizes the efficiency of information flow within local networks, and it reflects the ability of a network to tolerate faults^29^. The local efficiency of network *G* is computed as follows:

3 $${E}_{loc}(G)=\frac{1}{N}\sum _{i\in G}{E}_{glob}({G}_{i})$$

where *E*_*glob*_(*G*_*i*_) is the global efficiency of *G*_*i*_, the subgraph of the neighbors of node *i*.

#### Accuracy evaluation

We used accuracy to quantify the effect of scanning time on FC and network efficiency metrics. “Accuracy” was defined as the similarity strength between patterns from the relatively short and relatively long 10.0-min time series, as assessed using the Pearson correlation coefficient between the measure computed from the full-length time series and that computed from a given temporal window. For instance, for FC and the nodal efficiency metric, the Pearson correlation was calculated between group-level spatial maps from each short scanning window and that of the relatively longer 10.0-min scanning window. For local and global efficiency metrics, since there was only one number given to each network, the Pearson correlation between each short and relatively long temporal window was calculated across all subjects. Of note, the Fisher’s z-transformation was first conducted on each FC matrix before conducting the accuracy analysis.

#### Stability analysis

We also adopted a stability measure to examine the effect of scanning duration on FC and network efficiency metrics. “Stability” was defined as no significant differences between measures calculated from the relatively short and the relatively long 10.0-min time series^12^. For the FC metric, the measure was selected to be the correlation coefficient-associated standard deviation (SD). This is because the SD associated with the correlations of all nodal fNIRS time series provides a global measure of stability of the correlation values as opposed to the mean correlation values, which would merely approach zero. Specifically, the correlation coefficient-associated SD was generated as follows: the SD was first computed for each subject’s correlation coefficient matrix at each fNIRS signal acquisition duration. This generated 19 SDs (one per time bin) for each of the 53 subjects. The SD was then averaged across subjects at each time bin, leading to 1SD per time bin (19 time bins) for graphing. This enabled assessment of the changes in correlation coefficient variability (SD) as a function of the increasing fNIRS signal data collection duration. Subsequently, a one-way ANOVA was adopted to examine whether there was a significant difference between SD measures calculated from the relatively short and the relatively long 10.0-min time series. Post hoc analyses using the Dunnett test were also adopted to further examine significant effects revealed by ANOVA using the longest fNIRS signal acquisition duration (10.0 min) as the control group for pairwise comparisons.

A similar analysis was used for the stability evaluation of nodal efficiency. Of note, the nodal efficiency-associated standard deviation (SD) was selected to be the statistical measure in the statistical analysis. The calculation of the nodal efficiency-associated standard deviation was generated in a similar manner to the correlation coefficient-associated standard deviation (SD).

For network global and local efficiency metrics, a two-way ANOVA was conducted to explore the effect of fNIRS imaging acquisition duration on the magnitude of the computed network metrics at different sparsity values. The fNIRS signal acquisition duration (1.0~10.0 min in bins incrementally larger by 30 seconds) and sparsity (0.1, 0.2, 0.3, 0.4 and 0.5) were used as independent variables. Graph theory network metrics (local efficiency and global efficiency) were used as dependent variables. Notably, for local and global efficiency metrics, since only one number was given to each network, the statistical analysis between the full-length time series and a given temporal window was calculated across all subjects. Post hoc analyses using the Dunnett test were conducted to further examine significant effects revealed by ANOVA using the longest fNIRS signal acquisition duration (10.0 min) as the control group for pairwise comparisons.
